# Comparative Evaluation of Vision–Language Models for Detecting and Localizing Dental Lesions from Intraoral Images

**DOI:** 10.3390/jimaging12010022

**Published:** 2026-01-03

**Authors:** Maria Jahan, Al Ibne Siam, Lamim Zakir Pronay, Saif Ahmed, Nabeel Mohammed, James Dudley, Taseef Hasan Farook

**Affiliations:** 1 Department of Electrical and Computer Science, North South University, Dhaka 1229, Bangladeshsaif.ahmed02@northsouth.edu (S.A.); nabeel.mohammed@northsouth.edu (N.M.); 2 Department of CSE, National Institute of Technology Andhra Pradesh, Tadepalligudem 534101, India; 3 Adelaide Dental School, University of Adelaide, Adelaide, SA 5000, Australia; james.dudley@adelaide.edu.au

**Keywords:** staining, calculus, YOLO, deep learning, vision–language models

## Abstract

To assess the efficiency of vision–language models in detecting and classifying carious and non-carious lesions from intraoral photo imaging. A dataset of 172 annotated images were classified for microcavitation, cavitated lesions, staining, calculus, and non-carious lesions. Florence-2, PaLI-Gemma, and YOLOv8 models were trained on the dataset and model performance. The dataset was divided into 80:10:10 split, and the model performance was evaluated using mean average precision (mAP), mAP50-95, class-specific precision and recall. YOLOv8 outperformed the vision–language models, achieving a mean average precision (mAP) of 37% with a precision of 42.3% (with 100% for cavitation detection) and 31.3% recall. PaLI-Gemma produced a recall of 13% and 21%. Florence-2 yielded a mean average precision of 10% with a precision and recall was 51% and 35%. YOLOv8 achieved the strongest overall performance. Florence-2 and PaLI-Gemma models underperformed relative to YOLOv8 despite the potential for multimodal contextual understanding, highlighting the need for larger, more diverse datasets and hybrid architectures to achieve improved performance.

## 1. Introduction

Recent advances in artificial intelligence (AI), particularly computer vision, have demonstrated substantial potential to enhance dental diagnostics [1]. In computer vision, two principal methodological approaches are used: object detection models and vision–language models (VLMs) [2,3,4]. Object detection models are designed to identify, localize and classify distinct structures or lesions within an image such as carious lesions (tooth decay), non-carious lesions (including abrasion, attrition, or erosion) and calculus by delineating them with bounding boxes [5]. In contrast, VLMs offer advanced interpretive capacity by coupling visual analysis with language-based reasoning, thereby enabling broader contextual understanding of medical images [4,6].

The classification of intraoral images acquired directly from patients presents inherent challenges due to substantial variability in image quality, anatomical structures, and pathological presentations [7]. Inconsistent lighting, motion blur, and depth-of-field limitations impair clarity, while anatomical obstructions such as the tongue, saliva, and reflections from dental restorations further complicate visualization and, collectively, challenge AI-based classification. In addition, natural anatomical variations and artifacts such as food debris or metallic restorations may mimic pathology. The broad spectrum of intraoral findings, ranging from early-stage caries to periodontal disease with clinical attachment loss, significantly heightens diagnostic complexity. Distinguishing early non-cavitated carious lesions from non-carious lesions such as attrition remains particularly challenging in the absence of complementary data including radiographs or periodontal probing measurements.

Among object detection models, YOLOv8 (You Only Look Once, version 8), although not the newest in the YOLO family, remains widely adopted in research [8,9,10]. Previous studies have primarily focused on adjusting YOLOv8’s detection and attention mechanisms and optimizing its features for dental radiography [10]. Building on this, the current study extends YOLOv8’s application to complex intraoral photoimaging. This is implemented by partitioning images into grids and simultaneously predicting object location and class in a single step, it enables rapid and accurate identification of dental structures and disease features. To enhance contextual interpretation, contemporary computer vision systems increasingly integrate visual and textual information. PaLI-Gemma [11] and Florence-2 [12,13] represent this new generation of VLMs: PaLI-Gemma, developed by Google, combines language reasoning with image recognition, whereas Florence-2, developed by Microsoft, employs a unified multitask architecture to perform diverse visual tasks including segmentation, detection, localization, and captioning. Florence-2 demonstrates competitive performance in both fine-tuned and zero-shot settings and is capable of generating bounding boxes for multiple intraoral targets. 

With two major global leaders in AI research actively developing vision–language architectures, it is valuable to assess how their performance in detecting carious and non-carious lesions from intraoral photography. To the authors’ knowledge, this application has not yet been reported in the literature. Accordingly, this study compared the performance of two emerging VLMs, PaLI-Gemma and Florence-2, with YOLOv8, an established deterministic object-detection model, for the detection and localisation of dental lesions.

The following research questions were formulated:

1. How effectively can vision–language models identify and localize common dental lesions from intraoral photography, and how does their performance (Precision, Recall, and Mean Average Precision) differ across lesion classes?
2. Do multimodal foundation models (e.g., Florence-2, PaLI-Gemma) offer diagnostic advantages over deterministic object detectors in dental-imaging tasks, particularly with respect to contextual understanding and lesion classification?

## 2. Material and Methods

This study received ethical approval from the University of Adelaide Human Research and Ethics Committee (HREC-2023-073) and the Institutional Review Board of North South University (2023/OR-NSU/IRB/0503). The research was conducted in accordance with the Minimum Information about Clinical Artificial Intelligence Modeling (MI-CLAIM) checklist [14]. Additionally, all programming code adhered to the PEP 8 guidelines [15].

This section is structured as follows: an overview of the dataset (Section 2.1), preprocessing methods (Section 2.2), the models used (Section 2.3), and the evaluation metrics (Section 2.4).

### 2.1. Dataset Overview

The dataset comprises 174 de-identified intraoral photographs, including 90 images of the upper jaw and 84 images of the lower jaw, each with an approximate resolution of 3024 × 4032 pixels. All images were retrospectively obtained from a single specialist center that primarily managed referred dental cases predominantly undergoing complex full-mouth rehabilitation involved combinations of periodontal therapy, extensive restorations and extractions.

### 2.2. Data Pre-Processing

All images were de-identified at the source in accordance with the World Medical Association Declaration of Helsinki, with no treatments were altered. Therefore, individual written consent was not required. The photographs were annotated by a dentist with five years of experience in clinical machine learning using an open-source polygon-based annotation tool (LabelMe; wkentaro, https://github.com/wkentaro/labelme (accessed on 15 May 2025)) and exported as JSON files containing the annotation details through an end-to-end training platform (Roboflow; https://github.com/roboflow (accessed on 20 May 2025)). The dataset included the following annotation classes: staining, micro-cavitation, cavitation, calculus and non-carious lesions.

**Figure 1:**
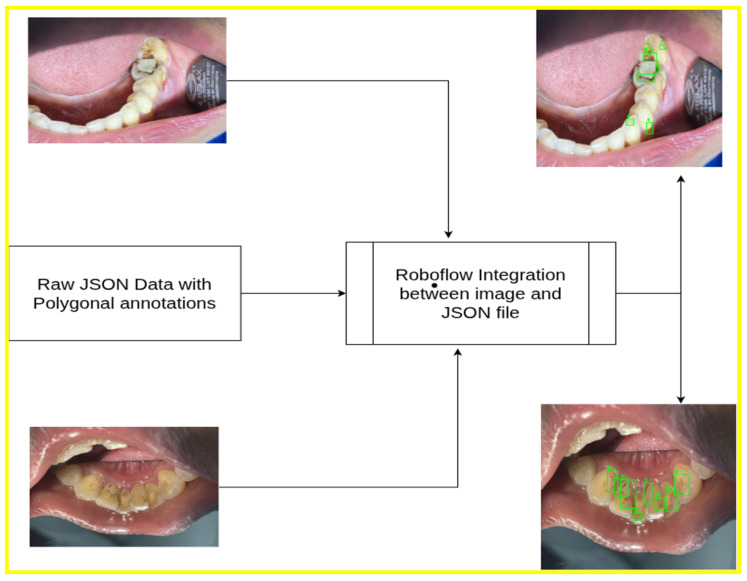
Dataset overview: polygonal JSON annotation of upper and lower jaw.

An overview of the processing workflow for upper and lower intraoral images, including annotation generation, is presented in Figure 1.

The experiments proposed were conducted in Google Colab and an associated T4 GPU. During pre-processing, the following steps were applied:

1. Auto-orientation: The images in the dataset were auto-oriented to correct any rotation inconsistencies.
2. Image resizing: All images were resized to a standardized resolution of 640 × 640 pixels to ensure uniformity during training and detection.
3. Augmentation: Data augmentation techniques were applied to improve model robustness. Three augmented outputs were generated with the following transformations:
  - Saturation was adjusted by +25% and −25%.
  - Brightness levels were modified from −2% to +2%.
  - Exposure was adjusted between −10% and +10%.

**Figure 2:**
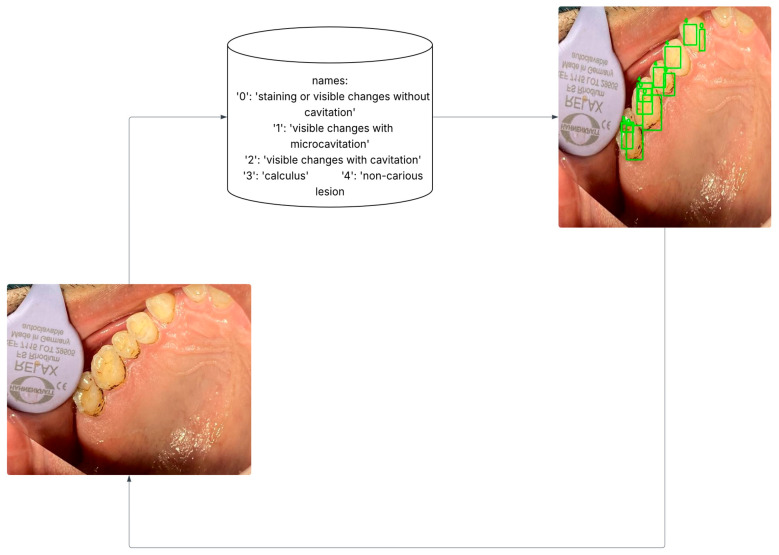
Data pre-processing: Transform polygonal annotation to oriented bounding boxes.

In YOLOv8, the selected classes were assigned numeric labels for streamlined processing: staining, micro-cavitation, cavitation, calculus and non-carious lesions. These classes and their corresponding codes are illustrated in Figure 2.

**Figure 3:**
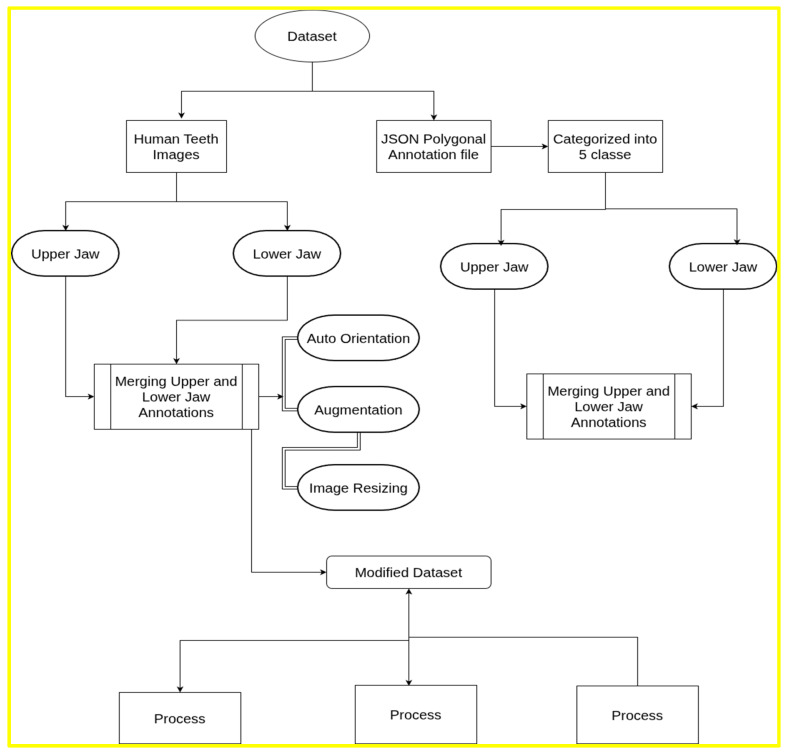
Dataset pre-processing and formatting pipeline.

The pre-processing approach used in YOLOv8 differed slightly from that used for the PaLI-Gemma and Florence-2 datasets. For the multimodal models, the data format incorporated an ‘image’ field specifying the annotated file name, along with two distinct fields (prefix and suffix) that define the beginning and end of the object-detection annotations, respectively. Both fields contained the identifier “<OD>,” which marked the start and end of the annotation block, thereby ensuring clear segmentation of object-detection data within each file. Figure 3 describes the data formatting pipeline followed for the current study.

Polygon-based annotations were transformed into minimal enclosing bounding boxes to enable compatibility with object detection frameworks. Although this method is widely used, it may reduce IoU-based metrics by introducing spatial imprecision for unevenly shaped lesions. This effect is more pronounced at stricter IoU thresholds and may partially contribute to decreased mAP value for vision models.

### 2.3. Model Architecture, Hyperparameter Tuning, and Workflow

#### 2.3.1. YOLOv8

The YOLOv8x variant, previously shown to achieve the highest mean average precision (mAP) (16), was employed in this study. The dataset was pre-processed and converted into the YOLOv8 bounding-box format. Model training was conducted on the designated training subset with an initial learning rate of 0.01; this rate was progressively reduced over subsequent epochs to support stable convergence. The model was trained for 100 iterations.

Model performance was evaluated using the test set comprising 16 images across five classes. A confidence threshold of 0.5 was applied, retaining only detections with a confidence score of 50% or higher.

#### 2.3.2. Florence-2

The study employed a single-architecture vision–language model capable of simultaneous object detection, segmentation and captioning within a prompt-driven, multi-task framework. Training was conducted with a batch size of four using a standard data loader. Fine-tuning was optimized using Low-Rank Adaptation (LoRA), which introduces trainable low-rank matrices into selected layers, thereby substantially reducing the number of parameters requiring training [16]. LoRA facilitates parameter-efficient fine-tuning by injecting low-rank updates into a subset of weight matrices while keeping the majority of pretrained weights frozen. In practice, only a small proportion of parameters is fine-tuned, typically a few percent depending on configuration. Additional implementation steps included: 1) setting input resolution, 2) applying augmentation strategies, and 3) performing domain-specific post-processing (e.g., class-mapping or regex-based label extraction) to align Florence-2 outputs with the target dental lesion classes [13].

In this experiment, LoRA layers were applied to selected target modules, including fully connected layers, convolutional layers, and output layers. To mitigate overfitting, a dropout rate was incorporated within the LoRA layers. The percentage of trainable parameters was calculated as follows:

$$trainable\%=(\frac{trainable\;params}{all\;params})\times 100$$

Substituting the values:

$$trainable\%=(\frac{1,929,928}{272,733,896})\times 100\approx 0.7076\%$$

**Figure 4:**
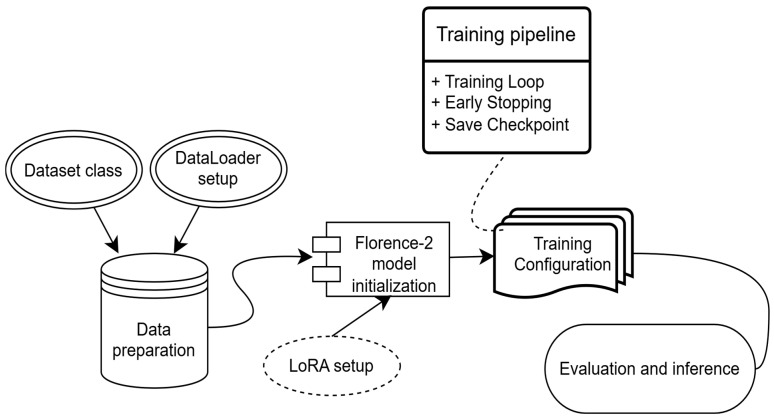
Experimental workflow for the Florence-2 model implemented for the intraoral image dataset.

Fine-tuning involved iterative training and validation, with regular-expression-based pattern matching used to extract class labels from model suffix outputs. The overall Florence-2 workflow is illustrated in Figure 4.

Florence-2 was fine-tuned using a parameter-efficient Low-Rank Adaptation (LoRA) method, with all backbone parameters frozen. Training was conducted for 1500 optimization steps, corresponding to approximately 20 epochs, using a batch size of 4. The AdamW optimizer was applied with an initial learning rate of 1 × 10^−4^;, weight decay of 0.01, and a cosine learning rate decay schedule.

Early termination was applied if validation loss failed to improve for five consecutive evaluation intervals to prevent overfitting given the limited dataset size. Gradient clipping was also used to stabilize training. Florence-2 converged consistently under this setup, although localization accuracy remained limited.

#### 2.3.3. PaLI-Gemma

PaLI-Gemma is a vision–language model that integrates Google’s Gemma language model for natural language understanding with the SigLIP image encoder for visual processing [17]. In this study, the PaLI-Gemma model with f16 precision and 3B parameters was used to optimize memory efficiency while preserving high accuracy.

The dataset comprised annotated files in JSONL format, with each line representing a single annotated sample. The five target classes were extracted from the prefix field in the first line of each annotation file. Bounding boxes and class labels were rendered onto the images for visualization. Each image was resized to 224 × 224 pixels to meet the input requirements of the SigLIP encoder.

During fine-tuning, the parameters of the language model were frozen, with updates restricted to selected layers to reduce storage demands. Training was performed with a batch size of two and a learning rate of 0.005, selected on the basis of preliminary experiments to balance stability and performance.

**Figure 5:**
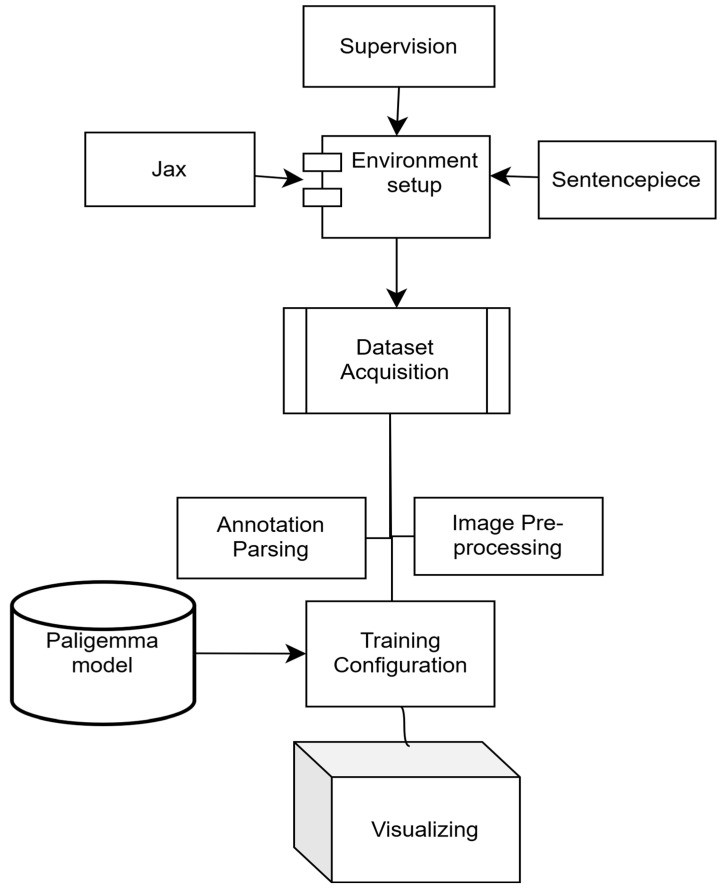
Experimental workflow for the PaLI-Gemma model.

The Big Vision repository, JAX, and related components were integrated to access pre-trained models, evaluation utilities and experimental tools. Additional dependencies, including compatibility packages for sentencepiece [18], were installed to ensure full functionality. The overall workflow is illustrated in Figure 5.

PaLI-Gemma was fine-tuned using a parameter-efficient strategy in which the majority of pretrained parameters were frozen and gradient updates were applied only to a subset of trainable parameters defined by a binary trainable mask. Training was conducted using a batch size of 2 on 114 annotated training examples, resulting in 57 optimization steps (one epoch).

Optimization was performed using stochastic gradient descent (SGD) with a manually applied parameter update rule. A cosine learning rate schedule with 10% warm-up was employed, with a base learning rate of 0.001. The training objective was an autoregressive token-level negative log-likelihood loss, computed only over valid tokens using a loss mask to exclude padding.

Training was intentionally limited to a single epoch to reduce overfitting given the small dataset size. No additional convergence-based early stopping criterion was applied; instead, training was terminated after the predefined number of steps. Model evaluation was performed at regular intervals using qualitative inspection and IoU-based detection metrics.

### 2.4. Evaluation Metrics

Object detection accuracy was evaluated using bounding box precision (Intersection-over-Union, IoU) [19], recall and mean average precision (mAP), which combines precision and recall into a single performance metric. The model was tested on randomly selected images from the test set to intuitively assess its generalization and learning capability. Two standard mAP metrics were used: mAP@50 (mAP at an IoU threshold of 0.50) and mAP@50–95 (the average mAP across IoU thresholds ranging from 0.50 to 0.95 in increments of 0.05). The mAP@50–95 is calculated as:

$$mAP_{50-95}=\frac{1}{10}\overset{0.95}{\underset{IoU=0.50}{\sum }}AP_{IoU}$$

The mAP@50–95 includes evaluation at higher IoU thresholds (e.g., 0.90), requiring predicted masks to overlap with ground-truth bounding boxes by at least 90%. In practice, dental imaging datasets typically produce lower mAP@50–95 scores due to high intra-class similarity, as teeth and lesions frequently exhibit visual overlapping.

The confidence threshold defines the minimum score (ranging from 0 to 1) required for a model to consider a predicted object valid [20,21]. Adjusting this threshold allows for evaluation of detection reliability: higher thresholds reduce false positives by filtering uncertain detections, while lower thresholds may increase the number of false positives [20]. To examine performance under different detection certainty levels, the model was evaluated using two confidence thresholds, 0.50 and 0.25.

For PaLI-Gemma and Florence-2, evaluation focused on mAP@50, mAP@50–95, and mAP@75, providing a detailed assessment of detection performance across multiple IoU thresholds.

## 3. Results

The current study compared the performance of Florence-2, PaLI-Gemma and YOLOv8 on the annotated oral dataset, analyzing metrics such as precision, recall, mean average precision (mAP), and class-specific performance at different confidence thresholds.

### 3.1. YOLOv8 Performance

YOLOv8 achieved a mAP@50 of 37%, at a confidence threshold of 0.25, which decreased to 35% when the threshold was raised to 0.5. Likewise, the mAP@50-95 values dropped from 18% at 0.25 to 17.7% at 0.5. These metrics indicate that a lower threshold allowed YOLOv8 to identify more instances, albeit with greater risk of false positives.

Precision and recall metrics also showed significant differences across thresholds:

- At a threshold of 0.25 (Table 1), the model achieved a precision of 42.3% and a recall of 31.3%.
- At a threshold of 0.5 (Table 2), precision increased to 54.7%, while recall dropped to 15.6%.

**Figure 6:**
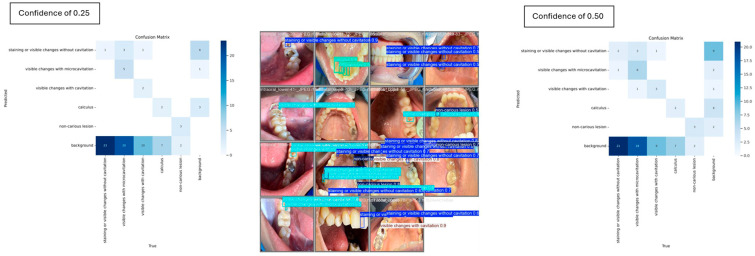
Individual misclassification distribution across the two confidence thresholds.

For the carious and non-carious lesion classes, YOLOv8 demonstrated strong performance in detecting visible changes associated with cavitation, consistently achieving 100% precision. However, at a confidence threshold of 0.5, the model completely failed to detect instances of the calculus class, yielding 0% mAP, precision and recall. When the threshold was reduced to 0.25, detection performance improved modestly, with the calculus class achieving an mAP of 16.7%. The pairwise confusion matrix distribution across the two thresholds is illustrated in Figure 6, highlighting the impact of threshold adjustment on class-specific detection outcomes.

**Figure 7:**
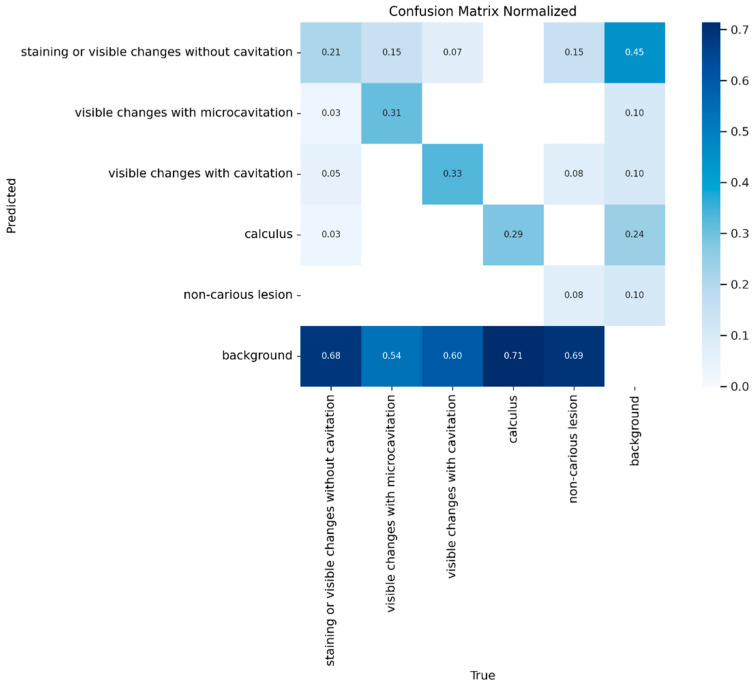
The normalized confusion matrix for predicted versus actual class distribution for YOLOv8.

The overall distribution of misclassifications across classes is shown in Figure 7, expressed as represented as the normalized ratio of predictions to ground truth.

### 3.2. Florence-2 and PaLI-Gemma

Florence-2 achieved an mAP@50 of 10%, mAP@75 of 3%, and an mAP@50–95 of 4%, indicating substantial difficulty in accurately localizing objects. Despite this, the model demonstrated moderately higher precision (51%) and recall (35%). PaLI-Gemma produced an mAP of 0%, indicating no overlap between predicted and ground-truth bounding boxes. The model achieved a precision of 13% and a recall of 21%, both of which were influenced by a high number of false positives, particularly where staining was incorrectly associated with other classes.

**Figure 8:**
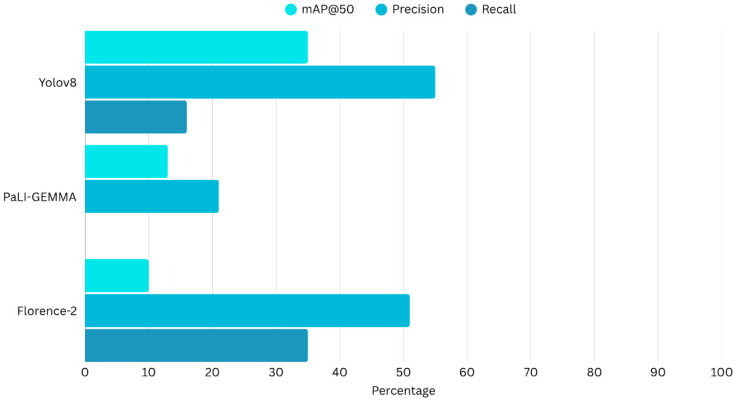
Comparison of the evaluation matrix of Yolov8, PaLI-Gemma and Florence-2.

Figure 8 presents a histogram comparing the three models and their overall diagnostic performance metrics.

## 4. Discussion

This study aimed to evaluate the effectiveness of VLMs in identifying and localizing common dental lesions from intraoral images. The unimodal object detection model YOLOv8 outperformed the multimodal models PaLI-Gemma and Florence-2 when applied to this small dataset. While all three models performed well for certain lesion categories, particularly those involving visible changes with cavitation, they struggled with more complex classes such as calculus. Detection of calculus remains challenging due to its highly variable appearance, which is influenced by factors such as diet and oral hygiene [22]. For example, smokers may present with calculus showing black tar-like deposits that can be misinterpreted as carious lesions, whereas bright yellow calculus may resemble non-carious lesions [22].

Although Florence-2 demonstrated higher precision and recall than YOLOv8 in certain cases, it was limited by low mAP scores and frequent confusion between stain, calculus and non-carious lesions. PaLI-Gemma failed to produce meaningful results, with no overlap between predicted and ground truth bounding boxes. These findings reinforce concerns that multimodal models require substantially larger, cleaner and more diverse datasets to achieve reliable performance. In smaller datasets, the inclusion of visually inconsistent classes such as calculus may further complicate differentiation between carious and non-carious lesions. While multimodal learning continues to evolve and is likely to improve over time, current VLMs remain insufficient for robust clinical image interpretation.

These results are consistent with existing literature demonstrating the effectiveness of YOLO-based architectures in medical imaging. Recent assessments of YOLOv8 highlight improved detection accuracy, localization precision and computational speed, making it particularly well suited for dental diagnostic applications [9]. Its anchor-free detection head and enhanced spatial pyramid pooling contribute to superior localization of small anomalies, which are frequently encountered in clinical imaging.

The findings suggest that, for noisy and complex intraoral imaging datasets, current VLMs remain in the early stages of developmental and are not yet suitable for reliable disease detection. At present, they are unable to meaningfully reduce human error in distinguishing carious from non-carious lesions using single intraoral photographs. VLMs tend to exhibit localized perceptual limitations, frequently missing subtle cues such as micro-calcifications and early enamel changes. Additionally, structural inconsistencies in their outputs can further undermine clinical reliability [23]. Collectively, these issues highlight the need for improved anatomical precision, domain-specific fine tuning and stronger integration of clinical reasoning within multimodal AI systems.

This study was limited by the relatively small dataset of 172 images, which may have constrained the models’ ability to generalize effectively. Small datasets pose significant challenges for large-scale models such as Florence-2 and PaLI-Gemma. Additionally, the dataset exhibited class imbalance, with certain categories, such as calculus, having fewer instances, contributing to uneven performance metrics. Variability in annotation quality, particularly the use of polygonal shapes, may also have influenced model training. This challenge was further compounded by the wide multispectrum appearance of calculus, which can vary substantially depending on individual dietary patterns. Given the limited size of the test set, the reported YOLOv8 performance metrics must be interpreted with caution. With only a small number of evaluation images and class instances, point estimates of precision, recall, and mAP are subject to statistical variability and may be sensitive to the specific composition of the test split. In such small-sample settings, confidence intervals are wide around these estimates which would theoretically reflect uncertainty rather than instability of the model itself. The present results are intended to offer an indicative comparison between models rather than definitive performance bounds. Formal interval estimation or bootstrapping in future research could provide a more precise quantification of this uncertainty.

Future studies should expand and diversify datasets, exploring additional forms of artificial data synthesis and pre-processing to improve training of VLMs on complex intraoral photographs. Further investigations could assess techniques such as synthetic data generation and advanced data augmentation, including geometric transformations, intensity adjustments, flipping and inpainting, to improve model generalizability [24]. Domain-specific fine-tuning of VLMs may also be helpful. For example, pretraining Florence-2, on curated intraoral datasets could improve performance on small, noisy or highly specialized imaging data. A potential strategy is to begin with simplified intraoral images containing singular or limited clinical findings and progressively increase complexity to enhance diagnostic capability over time [11]. A promising and achievable direction may be the development of hybrid models that combine the object-detection precision of YOLOv8 with the contextual understanding and multimodal strengths of Florence-2, supported by a transfer-learning pipeline built on intraoral datasets of increasing complexity.

Vision–language models (VLMs) are inherently high-capacity architectures designed to exploit large-scale multimodal pretraining in data-rich regimes. In small datasets such as the one used in the current experiment, these models exhibit low data efficiency because the number of task-specific samples is insufficient to effectively adapt to their large parameter space. Architecturally, VLMs rely on patch-based visual tokenization and transformer-based attention mechanisms that use global semantic alignment instead of spatial localization. As a result, fine-grained object detection tasks with irregular lesion boundaries and subtle visual differences are poorly constrained during fine-tuning. Even when parameter-efficient methods are applied, the effective model capacity remains disproportionately large relative to the dataset size affecting overall localization abilities.

The computational complexity of the evaluated methods varies significantly due to their architectural design. YOLOv8 is a single-stage object detection model whose inference complexity scales approximately as O(H·W·C), where *H* and *W* stand for the input image’s spatial resolution and *C* represents the number of convolutional channels. As YOLOv8 completes detection in a single forward pass without region recommendations, it enables near real-time inference and relatively low computational overhead.

Florence-2 and PaLI-Gemma-based systems showed higher computational complexity due to the integration of a large language model. The transformer-based self-attention mechanism, which scales as O(N^2^·D), where *N* is the number of visual tokens and *D* is the embedding dimension, dominates their inference complexity, while cross-modal attention and text generation further increase computational cost.

Although the dataset size limits the generalizability of VLM performance, the mAP of 0 observed for PaLI-Gemma cannot be attributed solely to data scarcity. Rather, it reflects the model’s difficulty in generating bounding boxes with sufficient spatial overlap to meet IoU-based evaluation criteria. PaLI-Gemma produced predictions with poor localization accuracy, resulting in false positives under mAP evaluation despite non-zero precision and recall. This finding suggests that, in their current form, vision–language models may be constrained in fine-grained medical object localization and lesion detection tasks.

## 5. Conclusions

The outcomes of the current study were:

- YOLOv8 achieved a mean average precision of 37% and a precision of 100% for cavitation detection. However, key limitations were observed, including low recall (~11%) for certain classes such as calculus, as well as reduced precision and recall at higher confidence thresholds. These reductions are likely attributable to aggressive filtering applied to smaller datasets with complex and overlapping annotation boxes.
- Florence-2 and PaLI-Gemma models underperformed relative to YOLOv8, given the dataset limitations, despite their potential for multimodal capabilities in contextual understanding, likely reflecting the need for larger, more diverse datasets or hybrid architectures to achieve improved outcomes.

## Figures and Tables

**Table 1:** YOLOv8 performance at 0.25 confidence threshold.

Classes	Images	Instances	Precision	Recall	mAP50	mAP50-95
All	16	79	42.3%	31.3%	37%	18%
Staining	16	24	21.7%	20.8%	15.3%	6.95%
Calculus	16	9	16.7%	11.1%	13.1%	6.55%
Micro cavitation	16	28	33.3%	21.4%	30.6%	7.43%
Cavitation	16	13	100%	23.1%	61.5%	29.1%
Non-carious lesion	16	5	40%	80%	64.6%	39.7%

**Table 2:** YOLOv8 performance at 0.5 confidence threshold.

Classes	Images	Instances	Precision	Recall	mAP50	mAP50-95
All	16	79	54.7%	15.6%	35%	17.7%
Staining	16	24	26.9%	8.33%	14%	8.4%
Calculus	16	9	0	0	0	0
Micro cavitation	16	28	96.5%	14.3%	48.6%	11.7%
Cavitation	16	13	100%	15.4%	57.7%	31.5%
Non-carious lesion	16	5	50%	40%	54.8	36.8%

## Data Availability

The data presented in this study are openly available in Github at https://github.com/maria-jahan20/Teeth-detection-using-VLM, last accessed on 26 November 2025.
